# Microsporidia *Nosema* spp. – obligate bee parasites are transmitted by air

**DOI:** 10.1038/s41598-019-50974-8

**Published:** 2019-10-07

**Authors:** Aneta Sulborska, Beata Horecka, Malgorzata Cebrat, Marek Kowalczyk, Tomasz H. Skrzypek, Waldemar Kazimierczak, Mariusz Trytek, Grzegorz Borsuk

**Affiliations:** 1 Department of Botany, University of Life Sciences, Akademicka 15, 20-950 Lublin, Poland; 2 Institute of Biological Basis of Animal Production; Faculty of Animal Sciences and Bioeconomy, University of Life Sciences in Lublin, Akademicka 13, 20-950 Lublin, Poland; 3 Laboratory of Molecular and Cellular Immunology, Hirszfeld Institute of Immunology and Experimental Therapy, Polish Academy of Sciences, Weigla 12, 53-114 Wroclaw, Poland; 4 Laboratory of Confocal and Electron Microscopy, Department of Biotechnology and Environment Sciences Centre for Interdisciplinary Research, John Paul II Catholic University of Lublin, Al. Kraśnicka 102, 29-718 Lublin, Poland; 5 Faculty of Biotechnology and Environmental Sciences, Centre for Interdisciplinary Research, Laboratory of Biocontrol, Production and Application of EPN, John Paul II Catholic University of Lublin, Konstantynów 1J, 20-708 Lublin, Poland; 6 Department of Industrial Microbiology, Institute of Microbiology and Biotechnology, Faculty of Biology and Biotechnology, Maria Curie-Skłodowska University, Akademicka 19, 20-033 Lublin, Poland

**Keywords:** Microbiology, Fungal biology

## Abstract

Microsporidia *Nosema* are transferred among bees via the faecal-oral route. *Nosema* spp. spores have been detected on flowers and transferred to hives along with the bee pollen. The aim of the present study was to determine whether *Nosema* microsporidia are transferred by air in an apiary, in a control area (without the presence of bee colonies), and/or in a laboratory during cage experiments with artificially infected bees. The novel way of transmission by air was investigated by the volumetric method using a Hirst-type aerobiological sampler located on the ground in the apiary, in the Botanical Garden and on the laboratory floor. Concurrently, the mean rate of *Nosema* infections in the foragers in the apiary was estimated with the Bürker haemocytometer method. Spore-trapping tapes were imaged by means of light microscopy, Nomarski interference contrast microscopy and scanning electron microscopy. The highest concentration of *Nosema* spores per 1m^3^ of air (4.65) was recorded in August, while the lowest concentration (2.89) was noted in July. This was confirmed by a Real-Time PCR analysis. The presence of *N. apis* as well as *N. ceranae* was detected in each of the tested tapes from the apiary. The average copy number of *N. apis* was estimated at 14.4 × 10^4^ copies per 1 cm^2^ of the tape; whereas the number of *N. ceranae* was 2.24 × 10^4^ copies per tape per 1 cm^2^. The results indicate that *Nosema* microsporidia were transferred by the wind in the apiary, but not in the Botanical Garden and laboratory by air. This was confirmed by genetic analyses. DNA from immobilised biological material was isolated and subjected to a PCR to detect the *Nosema* species. A fragment of the 16S rRNA gene, characteristic of *Nosema apis* and *N. ceranae*, was detected. Our research adds knowledge about the transfer of *Nosema* spp. microsporidia in the natural environment and indicates the season associated with the greatest risk of a bee colony infection with *Nosema* spp.

## Introduction

Microsporidia are ubiquitous in the environment and they infect almost all invertebrates and vertebrates^1^. The phylum Microsporidia is a large group of eukaryotic obligate intracellular parasites that can only complete their life cycle within an infected eukaryotic host cell^2^. Despite mitochondria that are reduced to mitosomes and a lack of some other organelles in the cell structure, microsporidia are acknowledged as belonging to the kingdom of Fungi^3,4^. *Nosema apis*^5^ and *Nosema ceranae*^6^ microsporidia are parasites of adult bees that are causing severe losses worldwide^7^. *N. apis* spores are 4–6 × 2–4 µm and *N*. *ceranae* spores are 3.3–5.5 × 2.3–3.0 µm in size^6,8^. The largest *N. ceranae* spores occupy a size range similar to that of the smaller *N*. *apis* spores^9^. Microsporidia are typically transmitted horizontally via the faecal-oral route, and an infection can occur, for example, by the ingestion of spores in feed via trophallaxis in the nest^10,11^. Sokół and Michalczyk^12^ found *Nosema* microsporidia in bee pollen and bee bread. However, during grooming of the body hairs^13–15^ and the formation of bee pollen, bees add droplets of nectar from the crop, which may contain these spores, to facilitate the gluing of pollen grains. In a honeybee colony, the foragers are potentially more likely to be exposed to infection than the hive workers^16–21^. Beshers *et al*.^22^ assumed that all foragers are older than any of the hive workers, and they are more infected by the microsporidia *Nosema*^18^. *Nosema* spores can also be transmitted indirectly via shared food sources or by the robbery of contaminated products^23–25^.

Infected *Lepidoptera*, namely the *Galleria mellonella* larvae which live in the hives, may disseminate the pathogen between colonies^26^. The transmission of *Nosema* may also be facilitated by *Merops apiaster*^27,28^ and *Loxostege sticticalis* adults which feed on flowers and may facilitate large-scale dispersals of the pathogen due to a high migratory activity^29^.

Previous research on *N. ceranae* infections and the deformed wing virus in wild bumblebee species^30–35^ has provided a basis for investigations of new routes for the spreading of pathogens that infect pollinating insects. Durrer and Schmid-Hempel^36^ have shown that shared flowers can lead to the horizontal transmission of *Crithidia bombi* among bumblebee colonies. This may be due to the risk of infection both in the nest and on the flowers, where the bumblebees leave their excrement while looking for nectar^36–38^. The similarity in the size of *Crithidia bombi* and *Nosema* suggests the same route of transmission for these parasites. Indeed, Graystock *et al*.^39^ identified *N. ceranae* in flowers*. Nosema* spores have also been found in bee pollen^12^. These findings prompted us to investigate the possibility of *Nosema* transmission in the air. The aim of the present study was to determine whether *Nosema* microsporidia are transferred by air in an apiary, in a Botanical Garden (BG; with no bee colonies), and/or in a laboratory during cage experiments with artificially infected bees.

## Results and Discussion

During the spore collection that occurred at the beginning of the experiment, the prevalence of uninfected colonies was 84%; whereas this value at the end of the experiment was 76%. The mean number of *Nosema* spp. per forager in the infected colonies increased from 7.4 × 10^6^ in June to 28.6 × 10^6^ in August (F_(29)_ = 22.96; p = 0.000014).

**Figure 1:**
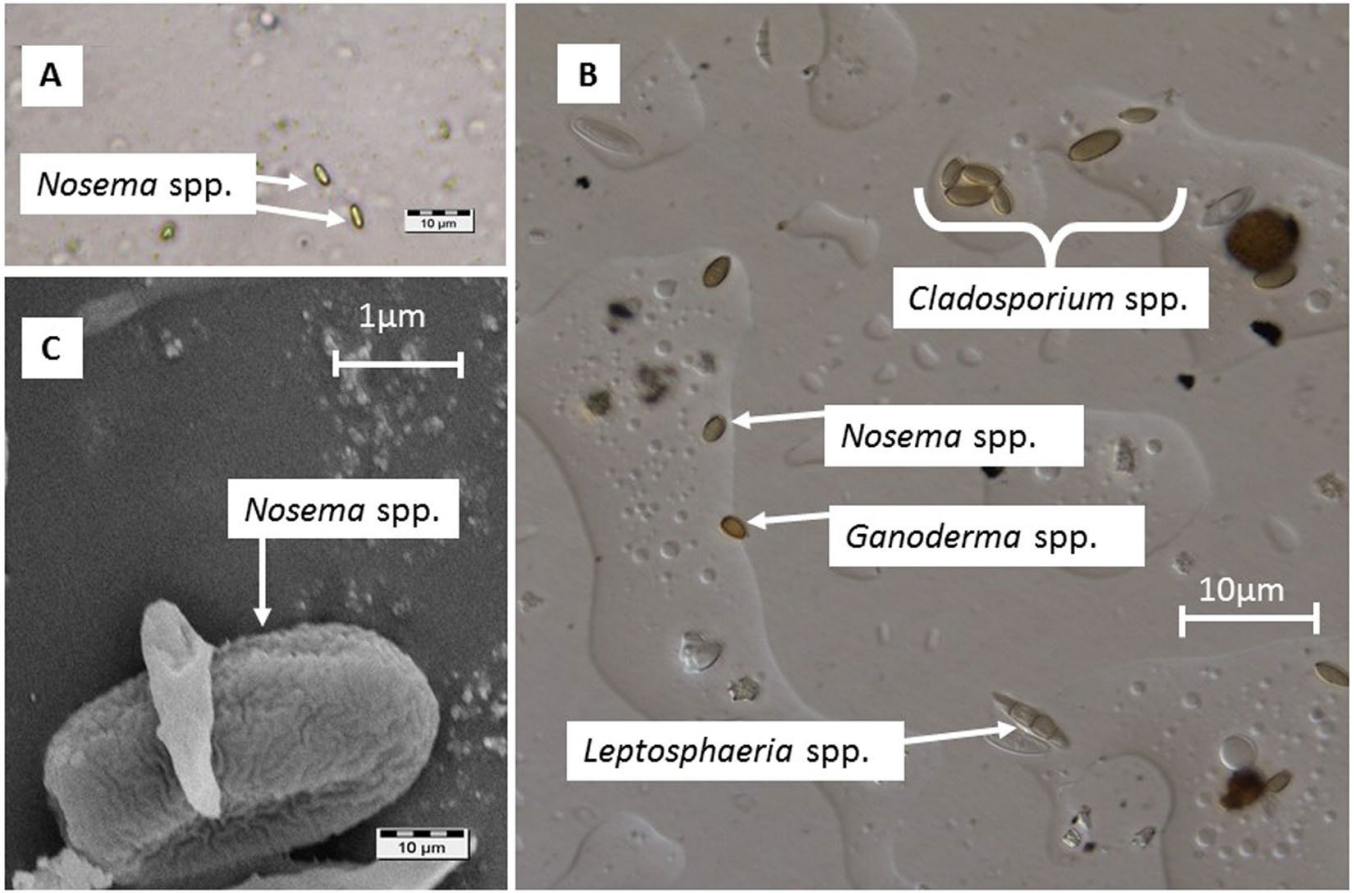
Microscope image. (**A**) Light microscope image of *Nosema* spp. spores on the tape. (**B**) Nomarski interference contrast microscope image of spores on the tape. (**C**) *Nosema* spp. spores on the tape visible under a scanning electron microscope.

The biological material, which was collected in parallel between June 1 and September 1 from the apiary air and immobilised on the sampler tapes, was analysed. Photographs were taken during the microscopic analyses of *Nosema* spp. observed with light (Fig. 1A) and with Nomarski interference contrast (Fig. 1B) microscopy. The *Nosema* spores were counted under the light microscope, as this was the easiest way to identify the spores on the collected tapes (Fig. 1A). Single or clustered *Nosema* spores were identified on the tapes among typical airborne fungi, such as *Ganoderma* spp., *Leptosphaeria* spp. and *Cladosporium* spp. (Fig. 1B). The highest concentration of *Nosema* spores per 1 m^3^ of air (4.65) was recorded in August, while the lowest concentration (2.89) was noted in July (F_(6)_ = 11.75; p = 0.029) (Table 1). There were no *Nosema* spores on the Hirst-type sampler tapes collected in the BG and in the laboratory (Table 1).

**Table 1:** Mean number of *Nosema* spp. in 1 m^3^ of air.

Location	Time Period		
Apiary	**June**	**July**	**August**
	3.45 ±0.55^ab^	2.89 ±0.11^a^	4.65 ±0.35^b^
Botanical Garden	0.00 ±0.00	0.00 ±0.00	0.00 ±0.00
Laboratory	**Week 1**	**Week 2**	**Week 3**
	0.00 ±0.00	0.00 ±0.00	0.00 ±0.00

± – standard deviation.

a,b – the different small letters denote statistically significant differences between the number of *Nosema* spores in the month (one-way ANOVA, F_(6)_ = 11.75, p = 0.029; Tukey test, p ≤ 0.05).

Although microscopic analyses are sufficient methods for the identification of spores in aerobiological studies, they would not provide conclusive scientific evidence in the present investigations due to the low morphological diversity of the *Nosema* spores. Therefore, we carried out an analysis of the spore-trapping tapes by a scanning electron microscopy, which showed the characteristic shape and sculpture of the outer structure of the *Nosema* cell wall (Fig. 1C)^39,40^.

**Figure 2:**
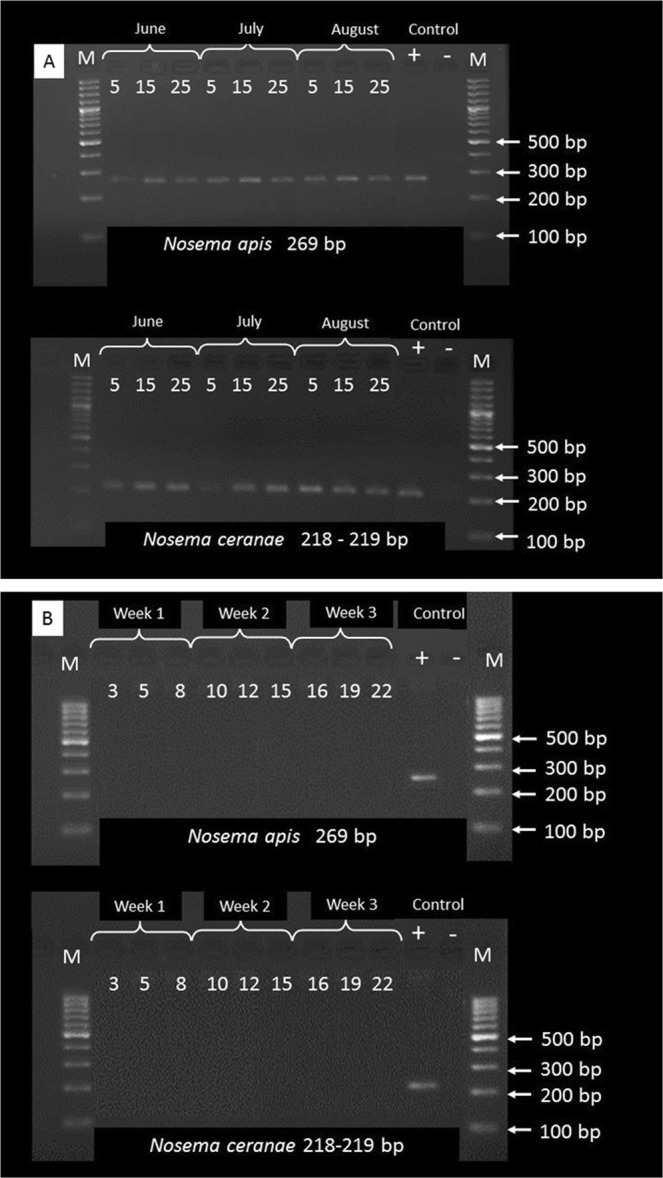
Experimental procedures. (**A**) 2% agarose gels showing PCR products amplified from *Nosema ceranae* and *Nosema apis* DNA extracted from the spores captured on apiary tapes. M: Molecular weight marker (Gene ruler, 1-kb ladder, Thermo Scientific). Lanes 5–25 correspond to the experimental days represented by the tape fragments selected for the PCR analysis. (**B**) 2% agarose gels showing absence of PCR products amplified from *Nosema ceranae* and *Nosema apis* DNA extracted from the spores captured on tapes from the air in the Botanical Garden. M: Molecular weight marker (Gene ruler, 1-kb ladder, Thermo Scientific). Lanes 3–22 correspond to the experimental days represented by the tape fragments selected for the PCR analysis.

In addition, molecular biology methods were applied to confirm our findings. The microsporidia immobilised on the tapes exposed to the apiary and the BG air were analysed by a PCR amplification of the 16S rRNA gene in order to determine the *Nosema* species (Fig. 2A,B). We detected amplicons corresponding to the expected size of the PCR products for *N. apis* (for 269 bp) and *N. ceranae* (218–219 bp) only in the apiary (Fig. 2A). By contrast, the amplification of the biological material trapped on the tapes exposed to the laboratory air resulted in no PCR products corresponding to the DNA of *N. apis* or *N. ceranae* spores. Identical results were obtained when the control tapes was exposed to the air in the BG, which was not influenced by the presence of honeybee colonies (Fig. 2B). The specificity of the PCR products was verified by Sanger sequencing, either directly (in the case of *Nosema ceranae*) or by employing a sub-cloning step (*Nosema apis*). The amplicons derived from *Nosema ceranae* were homogenous and were found to be an exact match to the rRNA gene sequences deposited in the public databases (NCBI) that were derived from several different strains of *N. ceranae* (NCBI accession number JQ639315.1). In the case of *Nosema apis*, by employing the sub-cloning step, we were able to reveal a relatively high level of diversity among the analysed amplicons: while all of the obtained sequences matched with regard to the *N. apis* rRNA gene, the level of identity ranged from 95.4% to 97% due to the occurrence of substitutions and 1-bp indels, which were most likely naturally occurring polymorphisms (NCBI accession number U97150). It is highly unlikely that the diversity among the analysed amplicons was a product of amplification errors, because identical reagents and procedures were used to amplify both the *N. apis* and *N. ceranae* DNA, and no such diversity was observed in the case of *N. ceranae*. The Real-Time PCR also confirmed the presence of *N. apis* and *N. ceranae* cells in each of the tested tapes from the apiary. The average copy number of *N. apis* was estimated at 14.4 × 10^4^ copies per 1 cm^2^ of the tape; whereas the number of *N. ceranae* was 2.24 × 10^4^ copies per tape per 1 cm^2^ (t_(16)_ = −2.518; p = 0.031) (Table 2).

**Table 2:** Quantitative analysis of the *N. apis* and *N. ceranea* genetic material in the tested tapes. The copy number was expressed as the average DNA copy number per 1 cm^2^ of the tape.

Tapes from the Apiary		Copy number × 10^4^		
Month	Days	*N. ceranae*	*N. apis*	Mean
June	5	2.60	44.4	17.9^b^
	15	2.51	52.1	
	25	2.27	3.38	
July	5	2.29	0.94	2.01^a^
	15	0.97	3.35	
	25	1.37	4.11	
August	5	1.90	9.08	5.12^b^
	15	4.40	7.31	
	25	2.82	5.22	
Mean		2.24*	14.4*	
Positive control		51 700	33 000	

*asterisks denote significant differences in the copy number per 1 cm^2^ of the tape between *N. ceranae* and *N. apis* (t-Student test, t_(16)_ = −2.518, p = 0.031).

a, b – the different small letters denote statistically significant differences between the copy number of *Nosema* spores per 1 cm^2^ of the tape in the month (one-way ANOVA, F_(15)_ = 16.438, p = 0.021; Tukey test, p ≤ 0.05).

*Nosema* microsporidia are obligate intracellular pathogens^41,42^ that infect honeybees and bumblebees^32^. Since biological objects measuring from 1 to 5 μm in diameter are classified as bioaerosols^43^, the 2−6 µm *Nosema* microsporidia^6,8^ can be classified as wind-dispersed bioaerosols. Bioaerosols are carried in the air and can be deposited on various surfaces in the natural environment, including on flowers^37,43^. The deposition of bioaerosols in the natural environment is influenced by both environmental (*e.g*. air currents, relative humidity and temperature) and physical factors (including the particle size, density and shape)^44,45^.

In our opinion, environmental factors can also influence the rate of deposition of *Nosema* spores (as bioareosols) and the time of their persistence on the surface, which may even contribute to a periodic increase in the concentration of spores in the air. This can be confirmed in the present study by the fluctuations in the number of spores immobilised on the tapes that occurred from the beginning of June to the end of August (Table 1). *Nosema* spp. can be spread on a large scale by *Merops apiaster* and *Loxostege sticticalis* due to a high migratory activity^27–29^.

We identified *Nosema* spores in the air of an open apiary field (containing 30 colonies), but we did not identify them in the BG, where there were no bee colonies within a radius of 4 km (Fig. 2B). This was probably due to the lower number of bees present in the air of the BG (Fig. 2B). The greatest number of spores per 1 m^3^ of air in the apiary was noted in August (Table 1). These results were confirmed by the Real-Time PCR analysis, which demonstrated a higher average number of DNA *Nosema* spp. copies in August (5.12 × 10^4^) than in July (2.01 × 10^4^) (F_(15)_ = 16.438; p = 0.021) (Table 2). Simultaneously, we observed a larger number of infected foragers in the bee colonies in August/September. August is the driest month in our latitude; therefore we believe that such conditions promoted the transfer of spores from fast-drying faeces by the wind and facilitated their spread in the air. During the spore collection that took place at the beginning of the experiment, the uninfected colony prevalence was 84%; whereas this value was 76% at the end of the experiment. The mean number of *Nosema* spp. per forager in the infected colonies increased from 7.4 × 10^6^ in June to 28.6 × 10^6^ in August (F_(29)_ = 22.96; p = 0.000014). The higher number of infected foragers in the apiary probably increased the concentration of spores in the air in August, compared with the level that was noted in early June; hence, we observed an increased number of spores trapped from the air (Table 1). *Nosema* spp. spores are transferred to the apiary air indirectly *via* the faecal-oral route^10,11,13,37^, thereby further increasing the risk of infection for individual bees and colonies (in an apiary consisting of 30 colonies).

Bigliardi and Sacchi^41^ suggested that spores present outside the host cells are metabolically inactive. *Nosema* spp. microsporidia generate environmental spores, which exhibit thermotolerance and a resistance to desiccation, and this plays an important role for epidemiology and for the laboratory studies of honeybee microsporidiosis^46,47^. However, at present there are no reports available on the capability of *Nosema* spores coming from the air to become infective after entering the bee’s digestive tract. An assessment of the infectivity of *Nosema* spores present in the air (i.e. those trapped on the tape) may not be feasible, as the tapes were coated with silicone oil, which can affect the viability of the spores.

Although we detected less than 5 *Nosema* spores in 1 m^3^ of air in the apiary containing 30 colonies (Table 1), an increase of this number to 72 spores per day (5 spores × 14.4 m^3^ of aerobiological sampler capacity) may increase the risk of infection for a single bee or a bee colony. It is difficult to compare this number with any other data, as there are no reports on the minimal number of spores responsible for causing infection in natural conditions. Bailey^48,49^ has found that one worker bee can be infected by a median infective dose below 100 spores of *Nosema apis*. In addition, Lotmar^50^ needed 1000 spores per bee to produce *N. apis* infections in 28% of the tested insects. However, McGowan *et al*.^51^ needed fewer spores to infect one bee. The median infective dose (ID50) was determined to be 149 *N. ceranae* spores per bee, and the minimum dose capable of causing a detectable infection was 1.28 spores.

Another example is the microsporidian *Vairimorpha necatrix* (Microsporida: Nosematidae) that infects *Trichoplusia ni* (Lepidoptera: Noctuidae), where infective doses as low as 11 spores have been shown to kill over 50% of the host larvae^52^. The infective dose of microsporidia depends on the species and its virulence, as well as on the age and physiological stage of a single host or the entire colony. Given the higher virulence of *Nosema ceranae* compared to *N. apis*^53,54^, and since the infection process may last for longer than 1 day^9^, 5 spores in 1 m^3^ seems to be a sufficient number to cause the infection of individual bees and the entire bee colony. Furthermore, it should be noted that the capacity of the sampler allowed for the analysis of only 14.4 m^3^ of air per day; however, more air passes through the apiary during the day, which may significantly increase the number of spores transferred to the bee colonies.

Microsporidia spores are commonly found in surface water (one of the sources of infection)^55^, but insects can also ingest the parasite during foraging, food processing or grooming^13–15^. Graystock *et al*.^37^ suggested that spores can be spread between the bee and the flower through the spore’s adhesion to the bee cuticle and subsequent rubbing them off onto the flower surfaces. Some parasite dispersal may be also associated with the formation of bee pollen and bee bread^12^. When the pollen is too dry, foragers add drops of nectar from the crop to glue the grains together and to form bee pollen^13–15,56^. The spores are then regurgitated along with the drop of nectar from the crop that may contain *Nosema* spores acquired from contaminated food. Since healthy and *Nosema*-infected honeybees visit the same flowering plants during their nectar and pollen collection, the spore transmission between the bee and the flower increases the risk of spreading *Nosema* to the colony. Such a phenomenon has been confirmed in the case of bumblebees. When bumblebees foraged on flowers that had been visited by honeybees, 23% of these insects, which were collected from the entrances of their hives, exhibited the presence of *N. ceranae*^37^.

The spores present on the honeybee’s body and on flowers are subject to drying under the influence of environmental factors (sun and wind). Thus, the small size (2−6 μm) of *Nosema* spp. spores^6,8^ enables them to be transferred as bioaerosols by wind in the air. To evaluate this possibility, the *Nosema* spp. spores were collected in the present study from the air in the apiary, where they were possibly dropped from the surfaces of the bodies of the foragers returning to the colonies together with the bee pollen^12^.

The foragers exhibited the highest rate of *Nosema* infection in August, i.e. in the period when we observed the greatest number of airborne *Nosema* spores. This indicates that the infected bees could generate the bioaerosol either during flights and defecation, or by transporting *Nosema* spores on their bodies, which were then blown away by the wind and deposited on the tapes of the Hirst-type trap (Table 1). All of the aforementioned routes of horizontal transmission of *Nosema* are highly possible ways of spreading the spores in any apiary^37,57–59^. Honeybees leave excrement near the apiary; then, when it is dry, the spores can be transported by the wind onto the anthers of blooming flowers and collected again along with the pollen^12^. We detected a higher number of copies of *N. apis* in comparison to *N. ceranae* in the tested tapes. This was consistent with results obtained by Copley *et al*.^60^, which suggest that *N. apis* is more abundant in samples of bottom scraps from infected colonies when compared to *N. ceranae*.

Honeybees are often housed in commercial apiaries containing tens to thousands of colonies. Thus, the high density of bees^61^ increases the horizontal transmission of diseases. The rate of parasite transmission between bees will predictably increase when the density of the pollinators in the air increases^62^. In addition, the increased competition for resources caused by the introduction of a high density of managed honeybees or commercially-produced bumblebees may also create stress for wild bees. Increased competition in foraging may have negative effects on some immune system components, impairing the resistance to parasites^63–68^. *N. ceranae* is an emergent honeybee parasite that is abundant at some sites but completely absent at others. Accordingly, *N. ceranae* has been implicated in the collapse of honeybee colonies in some, but not all, areas^7,10,17,54,69,70^.

As numerous studies on *Nosema*-infected honeybees are carried out in a laboratory using cage tests, we decided to check whether *Nosema* microsporidia will spread in the laboratory air. In the present study, we did not find *Nosema* spores in the laboratory air, presumably because the ventilation around the cages with bees was insufficient to lift the spores into the laboratory air. The low number of honeybees in the laboratory and the restricted space for flying in the cages limited the spread of the spores into the laboratory air; also, because the bees maintained in the cages could not fly, they did not collect pollen or excrete faeces. However, during the laboratory experiment, there were open ventilation holes in the bees’ cages, through which the *Nosema* microsporidia may have been transported into the laboratory air. Nonetheless, we did not detect the presence of *Nosema* microsporidia in the laboratory air. These results provide confirmation that experiments with both infected and non-infected honeybees can be performed in one laboratory.

## Conclusions

Our research adds knowledge about the transfer of *Nosema* microsporidia in the natural environment. Using the volumetric method with a Hirst-type sampler, we have shown a new route for the spreading of spores via the air in the investigated apiary. The results obtained in our apiary in one beekeeping season indicated the months associated with the greatest risk of a bee colony infection with *Nosema*. However, further investigations in other environments worldwide are required in order to evaluate the risk of the infection with *Nosema* in particular seasons. If our observations are confirmed in future studies, the proposed method will complement other methods of monitoring the levels of *Nosema* infection in apiaries. In our opinion, it could also have a potential application and provide useful information on air monitoring in areas characterised by a high density of crop plants before pollination. This is especially important in the case of wandering apiaries, which should be placed in *Nosema*-free areas.

## Materials and Methods

### Field experiment design

**Figure 3:**
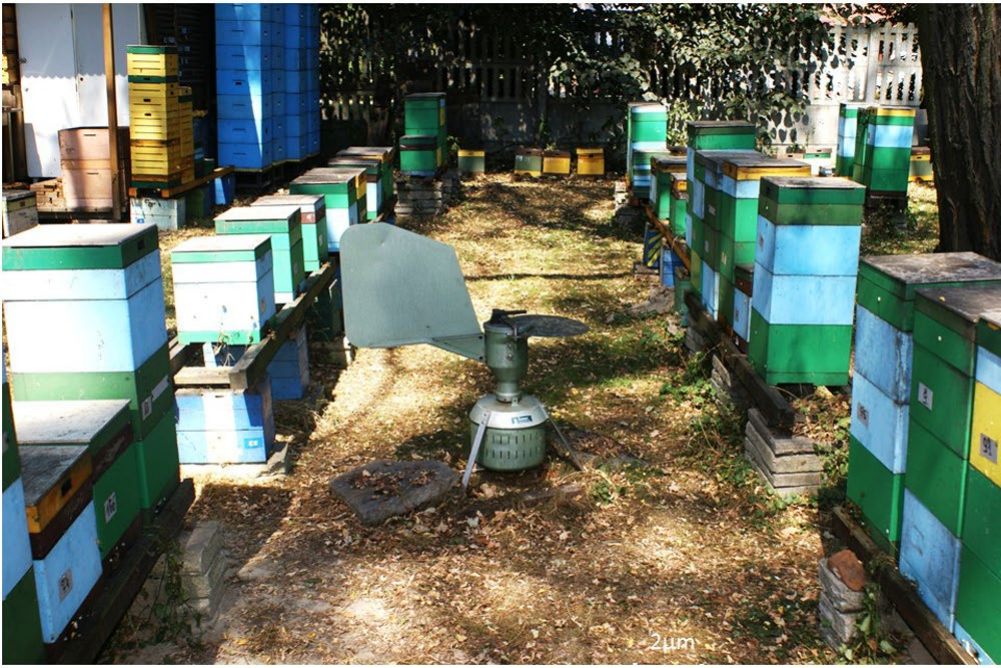
Experimental procedures. A Hirst-type spore trap was placed among the hives.

From 1 June to 1 September 2015, spores were collected from the apiary of the University of Life Sciences in Lublin (51°13′N, 22°38′E). Between 1 June and 1 September 2016, spores were also collected from the Botanical Garden (BG) of the University of Maria Curie-Skłodowska (51°26′N, 22°51′E) without the presence of bee colonies within a radius of 4 km, which was a control relative to the spores collected from the apiary. The spores present in the air were monitored by the volumetric method using a Hirst-type sampler (Burkard Manufacturing Company Ltd.) placed between the rows of hives (Fig. 3). On 1 June, immediately after installing the Hirst-type sampler, the mean rate of *Nosema* spp. infections in foragers and the prevalence of infections in the apiary were estimated with the microscopic method^71^. Briefly, 30 bees from each of the 30 colonies were homogenised in 30 mL of distilled water. The homogenous suspension was then placed in a Bürker haemocytometer. The spores in each square were counted under a light microscope at 400× magnification and were expressed as the number of spores per honeybee. On 1 September, after the spore trapping from the air had ceased, the foragers in the bee colonies were again examined to detect the presence of *Nosema* spores.

### Laboratory experiment design

Cage tests were carried out with infected bees and spores, which were trapped in the laboratory air for examination in the laboratories at the University of Life Sciences and at the University of Maria Curie-Skłodowska in Lublin. Brood combs were taken on the 20th day of development, which were placed in an environmental chamber, and maintained at a constant temperature (34 °C) and relative humidity (60%) to obtain 1-day-old honeybees. The cage tests were performed in 60 wooden cages (12 × 12 × 4 cm) equipped with glass screens on the front and having ventilation holes. There were no combs or wax foundations in the cages. The experimental groups comprised 1-day-old bees that were maintained in laboratory conditions (25 °C; H = 65%). On the 3rd day after emerging, the honeybees were inoculated by mass provisioning with a solution of *N. ceranae* and *N. apis* spores (5 × 10^6^ spores/mL)^72^, which were previously purified by single centrifugation (20,000 g; 30 min) in the Percoll gradient^73^. The pellets of *Nosema* spp. obtained were re-suspended in sugar syrup (1:1). Subsequently, the honeybees in the cages were provisioned with the spore suspension over three days, and were then maintained in the laboratory room for another 19 days. The average quantity of spores (5.25 × 10^5^) administered to each bee was estimated from the total volume (4.2 mL) of the spore-containing syrup consumed by all the bees in a cage.

Spores were collected between 1 June and 22 June 2017 (Burkard Manufacturing Company Ltd.), with a Hirst-type sampler which was placed at the height of the ventilation holes in the bee cages. We applied the volumetric method to monitor the spores as described for the apiary and BG tests.

### *Nosema* spp. trapping method

Before the experiments for monitoring *Nosema* spores in the air with the Hirst-type spore trap (which is used in aerobiological studies), a blind probe was conducted. The aim was to determine whether faecal particles were deposited on the sampler tapes collecting the spores present in the air. The probe operated by placing the faeces near the air inlets of the sampler located in the BG, where there were no bee colonies within a radius of 4 km. The macro- and microscopic analysis, which was performed after 7 days of exposure, confirmed the absence of faecal lumps on the tape.

This allowed us to conduct an experiment in which the spores were monitored using the volumetric method. The Hirst-type spore trap (Burkard Manufacturing Company Ltd.) was placed at ground level in the apiary and the BG and on the floor in the laboratory^74^. The trap operated continuously with a negative pressure at 0.01 m^3^ (10 L) min^−1^ (14.4 m^3^ of air per day) through a critical orifice (14 × 2 mm) facing the air flow with the use of a wind vane. The air flow was provided by an external vacuum pump. A rotating drum, to which particles adhere inside each trap, was driven by a clockwork mechanism that moved at a constant speed of 2 mm h^−1^ (48 mm per day) and completing one full rotation per week. The drum was covered with approximately 345 mm of Melinex tape (48 mm per day × 7 + 9 mm for mounting the tape) coated with silicone oil, which has been suggested for use in varying weather conditions^75^. Particles in the air flow were impacted on the tape due to the inertia of momentum^76^. The tape was changed once a week at the same hour of the day (9 AM) and was divided on a cutting block into seven equal segments corresponding to each sampling day^77^. Each tape segment with trapped spores was cut lengthwise into two fragments. One segment was used to make glycerine jelly preparations^74^, which were analysed afterwards using a Nikon light microscope (×400 along two horizontal lines, 48 mm long) (Fig. 1A), a Nomarski interference contrast microscope (Fig. 1B) and a scanning electron microscope (Fig. 1C). The other fragment of the tape was stored at −20 °C until the DNA extraction was performed. Nine tape fragments were randomly selected from the apiary and BG tapes (three fragments from the tapes representing Days 5, 15 and 25 were selected for each month of the spore collection). We also selected nine tape fragments randomly from the cage tests (these tape fragments represented Days 3, 5 and 8 in Week 1; Days 10, 12 and 15 in Week 2; and Days 16, 19 and 22 in Week 3). The number of *Nosema* spores from all tapes was multiplied by a correction factor to estimate the number of spores on the entire microscope slide. The results were expressed as the average daily concentration of spores in 1 m^3^ of air.

### DNA extraction from spore samples, PCR, Real-Time PCR and sequencing conditions

Lysis buffer (180 μL) and proteinase K (20 μL) were added to each tape fragment for 24 h, and the total DNA was isolated using the DNeasy Blood and Tissue Kit (Qiagen) in accordance with the manufacturer’s instructions. To identify the *Nosema* species, the total DNA was analysed by PCR using the 218MITOC (FOR 5-CGGCGACGATGTGATATGAAAATATTAA) and 321APIS (FOR 5-GGGGGCATGTCTTTGACGTACTATGTA) primers as described by Martin-Hernandez *et al*.^78^. The PCR was performed using the Taq PCR Core Kit (Qiagen). In a total volume of 30 µL, the reaction mixture included 6 µL of the DNA sample, 1 × PCR buffer, 1 × Q solution, 4 mM of MgCl_2_, 0.2 mM of each dNTP, 0.4 µM of each primer and 2 U of Taq DNA polymerase. The PCR conditions were as follows: 10 min at 95 °C, followed by 35 cycles of 30 s at 95 °C, 30 s at 55.8 °C, and 45 s at 72 °C, with a final extension at 72 °C for 7 min. The PCR products were electrophoresed on a 2% agarose gel and analysed under a UV light.

To additionally confirm the presence of the *Nosema* species representatives in the tested tapes, a Real-Time PCR analysis was carried out. Two pairs of primers were used in the reaction - NosAF–5-CATGTCTTTGACGTACTATGTACT and NosAR–ACTAGCTGATAGGTCTCACTCT for the detection of *Nosema apis* (product length 132 bp), as well as NosCF–5-ACGTAATACGATCAGATGGTCAGC and NosCR–5-CTCGAACGAAATGTCCCATCA for the detection of *Nosema ceranae* (product length 168 bp). To avoid non-specific amplification, the primer NosCF contains two mismatched nucleotides at the 3′ end, which prevent hybridisation with the *N. apis* template. NosAF was designed for a region that contains five mismatched nucleotides, which prevent hybridisation with the *N. ceranae* template at the 3′end. Both of the primer pairs were first tested in a conventional PCR to confirm the amplification of only the specific target. Specificity was confirmed by sequencing the amplification products.

The Real-Time PCR was prepared using 1 × PowerUp*™* SYBR*®* Green Master Mix (Thermo Fisher Scientific), 0.2 µm of NosAF and NosAR primers (master mix for the detection of *N. apis*), and 0.15 µm NosC and NosCR primers (master mix for the detection of *N. cerenae*) in a total volume of 20 µl in the 7500 Fast Real-Time PCR System. The cycling conditions were as follows: 2 min at 50 °C (UDG activation) and 2 min at 95 °C, followed by 40 cycles of 3 s at 95 °C and 30 s at 60 °C. A quantitative determination was performed in order to evaluate the average number of copies of *N. apis* and *N. ceranae* in the tested samples. Serial dilutions of 1 × 10^7^ copies/μl, 1 × 10^6^ copies/μl, 1 × 10^5^ copies/μl, 1 × 10^4^ copies/μl, 1 × 10^3^ copies/μl, 1 × 10^2^ copies/μl and 1 × 10^1^ copies/μl were used to prepare the standard curve. These dilutions were prepared using purified *PCR* products with known concentrations. Positive and no template controls were run with each plate. The products of the amplifications were separated on 2% agarose gel to exclude the presence of primer-dimer structures and non-specific products. The copy number was expressed as the average copy number per 1 cm^2^ of the tape.

The specificity of the PCR products was verified by the Sanger sequencing of the amplicons excised from the agarose gel. The *Nosema ceranae* rRNA gene amplicons were sequenced directly, whereas the *Nosema apis* amplicons were cloned prior to sequencing. The PCR products were cloned in the pGEM-T Easy vector (Promega) and transformed into *E. coli* cells, which were subjected to blue/white screening. Several individual plasmids isolated from the white colonies were then sequenced using T7 (5′-TAATACGACTCACTATAGG) or SP6 (5′-ATTTAGGTGACACTATAG) primers. The analysis was performed in triplicate. The obtained sequences were checked against the public nucleotide databases (NCBI) and were then submitted under accession numbers MG770412 to MG770420.

### Scanning electron microscopy

To confirm that the spores observed on the tape under the light and Normarsky microscopes were *Nosema* spp. spores, the same sections of the tapes with the trapped spores were prepared for a scanning electron microscopy (SEM). Briefly, the tapes were fixed for 24 h in 5% glutaraldehyde (v/v) in a 0.1 M phosphate buffer (pH 7.3). After fixation, the samples were washed in saline and dehydrated in an alcohol series, then dried in a critical point dryer (CPD7501, Polaron Range) and sputter-coated (SC 7620, Polaron Range) with an Au/Pd layer. The samples were examined in a LEO 1430 VP SEM, as well as being measured and photographed.

### Statistical analysis

The statistical analysis was performed using SAS software Version 9.5 (Statistical Analysis System Institute, Cary, NC). The numbers of *Nosema* spp. per forager in the infected colonies from June to August were compared using a one-way ANOVA, Tukey test. Differences of p ≤ 0.05 were considered significant. The DNA copy numbers per 1 cm^2^ of the tape were compared for *N. ceranae* and *N. apis* using the t-Student test. The number of spores and the DNA copy number of *Nosema* spp. identified on the tapes from June to August were compared using the one-way ANOVA, followed by Tukey test. Differences of p ≤ 0.05 were considered significant.
